# Daily Emotional Dynamics of Custodial Grandfamilies: Cross-Dyad Correlations of Affect Means and Variability

**DOI:** 10.1093/geroni/igab046.316

**Published:** 2021-12-17

**Authors:** Saul Castro, Frank Infurna, Britney Webster, Gregory Smith, Max Crowley, Megan Dolbin-MacNab, Carol Musil

**Affiliations:** 1 Arizona State University, Arizona State University, Arizona, United States; 2 Arizona State University, Tempe, Arizona, United States; 3 Kent State University, Kent, Ohio, United States; 4 Pennsylvania State University, University Park, Pennsylvania, United States; 5 Virginia Polytechnic University, Blackburg, Virginia, United States; 6 CWRU School of Nursing, Cleveland, Ohio, United States

## Abstract

Evidence indicates that daily emotional dynamics are associated with mental and physical health. However, these processes have not been examined among custodial grandmothers taking care of adolescent grandchildren. This daily diary study examined correlations between grandmothers and adolescents’ mean levels and variability in negative (NA) and positive affect (PA). Custodial dyads (M = 214) across the nation completed two weeks of daily surveys. For both grandmothers and adolescents, their own PA means were negatively correlated with NA means, PA variability, and NA variability; NA means were positively correlated with variability in PA and NA (ps <.01). Across dyads, grandmothers’ PA means were positively correlated with adolescents’ PA means and negatively correlated with adolescents’ NA means, PA variability and NA variability. Grandmothers’ NA means were positively correlated with adolescents’ variability in PA and NA (ps <.01). Our findings demonstrate how daily emotional dynamics are correlated within and between family members.

